# Seven Year Decline of Mountain Hare Abundance in the Peak District, England

**DOI:** 10.1002/ece3.71131

**Published:** 2025-03-17

**Authors:** Carlos P. E. Bedson, Katherine Walsh, Humphrey Q. P. Crick

**Affiliations:** ^1^ Natural England, Eastbrook Cambridge UK; ^2^ Department of Natural Sciences Manchester Metropolitan University Manchester UK

**Keywords:** blanket bog, conservation, distance sampling, extinction, grouse moor, mountain hare, population cyclicity

## Abstract

In England, the mountain hare is found only in the Peak District and is a remnant population surviving from translocations from Scotland during the 1870s of the genetically distinct subspecies *Lepus timidus scoticus* . Population monitoring undertaken by Manchester Metropolitan University and Queen's University Belfast began in 2017, continuing to 2021. Subsequently, monitoring was conducted by Natural England during 2022–2024, reporting for two sites, Bleaklow and Margery Hill, previously shown to have the highest densities of mountain hares. Standardised survey and analytical techniques remained identical to previous monitoring, enabling evaluation of change in absolute estimates of hare density from 2017 to 2024. Using distance sampling analysis, results showed a statistically significant decline of density from 2017 = 15.9 hares km^−2^ (95% CI 10.4–24.4) to 2024 = 6.7 hares km^−2^ (95% CI 3.9–11.5) (*p*‐value = 0.0001). When stratifying by habitat class, the highest densities of hares km^−2^ were found on restored blanket bog (27.9) and were statistically significantly higher than all other surveyed habitat classes: acid grassland (9.0), grouse moor bog (9.3), grouse moor heather (8.3), unrestored bog (19.3) and unmanaged dwarf shrub heath (4.0). All habitat classes showed declines; the largest were on grouse moors. For the two combined survey sites, the 2024 absolute population abundance was estimated at 542 individuals (95% CI 315–929). Extrapolating to the wider Peak District, the whole population abundance was estimated to have declined from 3562 individuals (95% CI 2291–5624) to 1038 individuals (95% CI 604–1765). Were this trajectory to continue, the mountain hare would likely disappear from England within the next few years. The causes of decline since 2017 are unknown. Lagomorph population sizes can fluctuate substantially owing to parasite mechanisms. Mountain hares persist better on restored blanket bog areas. Overall, the population is small and declining, exhibiting increased extinction risk and meriting conservation.

## Graphical abstract

From 2017 to 2024, systematic transect surveys of the only mountain hare population in England showed a statistically significant continual decline of 58%. It is not known whether this decrease is symptomatic of parasite mediated population cyclicity or other factors. The whole population estimate stands at 1038 individuals, and with the declining trajectory, it shows an increasing extinction risk, meriting continued conservation.

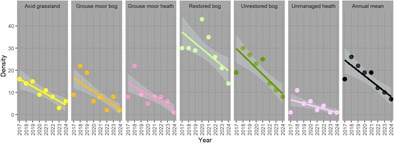

## 1 Introduction

The mountain hare ( *Lepus timidus* ) evolved within the genus *Lepus* during the Pliocene (Waltari and Cook 2005). Today, 16 subspecies are recognised, based on different morphologies and genetics, occupying cold climates at high latitudes or elevations across northern regions of Europe and Asia (Angerbjorn and Schai‐Braun 2023). Mountain hares are important herbivores: they inhabit forests or tundra, browsing *Betula*, *Salix* and *Picea* and other shrub habitats such as the heather moorlands ( *Calluna vulgaris* ) in Scotland. They recycle seeds and nutrients and are prey species for carnivores and raptors (Harris and Yalden 2008). Populations fluctuate substantially, caused by parasites, disease, predators, starvation and adverse weather events (Smith and Johnston 2019).

**FIGURE 1:**
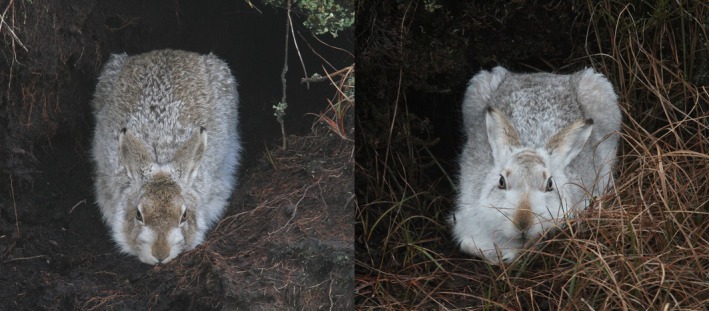
Two photos taken by the author of mountain hares( *Lepus timidus* ) at Bleaklow, Peak District UK, 29 January 2025, clearly showing on the same day two different pelage manifestations: Browner on the left, whiter on the right. In Scotland, Zimova et al. (2020) evidenced a lack of evolutionary shift in moult phenology in the context of climate change. Within the Faroe Island population, Giska et al. (2019) confirmed a winter‐grey variant arising from introgression by a single non‐colour changing founder.

In recent times, the mountain hare experienced substantial abundance and range reductions over its distribution, including Ireland, Scotland, Sweden, Russia, and the Alps, arising from competition or hybridisation with European brown hares ( *Lepus europaeus* ), hunter harvest and land use change (Angerbjorn and Schai‐Braun 2023). Mountain hares symbolise climate change effects: a cold‐preferring high elevation species, lacking a sufficiently swift change in moult phenology, thus experiencing white pelage camouflage mismatch against decreasing snow cover (Figure 1) (Zimova et al. 2020). Mountain hare pelage may itself vary; for example, Giska et al. (2019) evidenced a winter‐grey variant in the Faroe Islands.

In Great Britain, mountain hares reached Scotland from Scandinavia after the last glacial maximum, developing to become the subspecies *Lepus timidus scoticus* , experiencing a population bottleneck ~500 generations ago, genetically distinct, with low diversity compared to other subspecies (Hamill et al. 2006). The contemporary mountain hares of England migrated northwards following the glacial retreat 6000 BP (Yalden and Barrett 1999). During recent centuries, mountain hares in northeast Scotland benefitted from grouse moor heather habitat management, in some areas achieving high abundance ~200 individuals km^−2^ (Watson and Hewson 1973). During the 1870's mountain hares ( *Lepus timidus* ) were translocated from Scotland to the uplands of the Peak District, England, by sporting landowners; one of just a few successful reintroductions anywhere in Europe (Smith and Johnston 2019). The mountain hare became an important herbivore for the upland ecosystem, potentially genetically distinct, popular with conservationists and tourists, and emblematic of the Peak District.

The UK conservation status of mountain hares is periodically assessed and reported under the Conservation of Habitats and Species Regulations 2018, Section 9A. In Scotland, the mountain hare population was estimated at ~132,000 individuals (95% CI: 79,000–516,000) (JNCC 2019) and here the species is fully protected on Schedule 5 of the 1981 Wildlife and Countryside Act, which prohibits the intentional killing, injuring, or taking of the species as well as their possession and trade. In England, the mountain hare persists with a small population estimated at ~3,500 individuals (95% CI: 2200–5600) (Bedson et al. 2022), separated from those in Scotland by greater than 200 km, exceeding dispersal distances (Angerbjorn and Schai‐Braun 2023). Recommendations to support a change in the protection of mountain hares in England were submitted to the 7th Quinquennial Review process. This process, which takes place every 5 years, reviews the status of protected species on the Act.

For conservation assessments in the Peak District, a variety of mountain hare population surveys consisting mostly of field observation counts occurred intermittently from the 1950's to 2001 (Mallon et al. 2003). From 2017 to 2021, standardised systematic research was conducted by Manchester Metropolitan University and Queen's University Belfast (Bedson et al. 2022) mostly upon two sites, Bleaklow and Margery Hill. Here, the highest hare densities were found on blanket bog ecologically restored for carbon sequestration (32.6 individuals km^−2^). Fewer hares were reported on land managed for red grouse ( *Lagopus lagopus* ) shooting (12.2 individuals km^−2^ on grouse moor bog; 10.0 individuals km^−2^on grouse moor heather); an estimate considered far lower than that of Scottish grouse moors approaching ~200 individuals km^−2^ (Harris and Yalden 2008). Density on acid grassland used for sheep farming was also found to be modest (11.8 individuals km^−2^).

Mountain hare population monitoring continued in 2022–2024, conducted by Natural England, the UK government's advisor on conservation in England, repeating surveys at Bleaklow and Margery Hill. This paper adds to the previous 2017–2021 research.

Here we assess eight years' of monitoring data and report estimates of a declining trajectory of mountain hare density by habitat class. We consider how this survey scheme compares to ‘best practice’ for wildlife monitoring methodologies. We contemplate whether these findings represent natural population cyclicity, consider factors that may contribute to extinction risk, and the importance of mountain hares to conservation.

## 2 Methods

This research consistently follows methods established by Bedson et al. (2022), enabling comparable and transparent reporting of mountain hare densities across the studies of 2017–2021 (Bedson et al. 2022) continuing 2022–2024 (this paper). We deliberately reproduce below, almost verbatim, the methodology from Bedson et al. (2022) sections “2.1 Study area”, “2.2 habitat classes”, “2.3 Surveys”. Section “2.4 Distance modeling” is identical to the approach described in Bedson et al. (2022), yet adds 2022–2024 data, and re‐evaluates previous analytical choices. This repetition of methods, helps readers see that approaches were identical throughout 2017–2024.

### 2.1 Study Area

**FIGURE 2:**
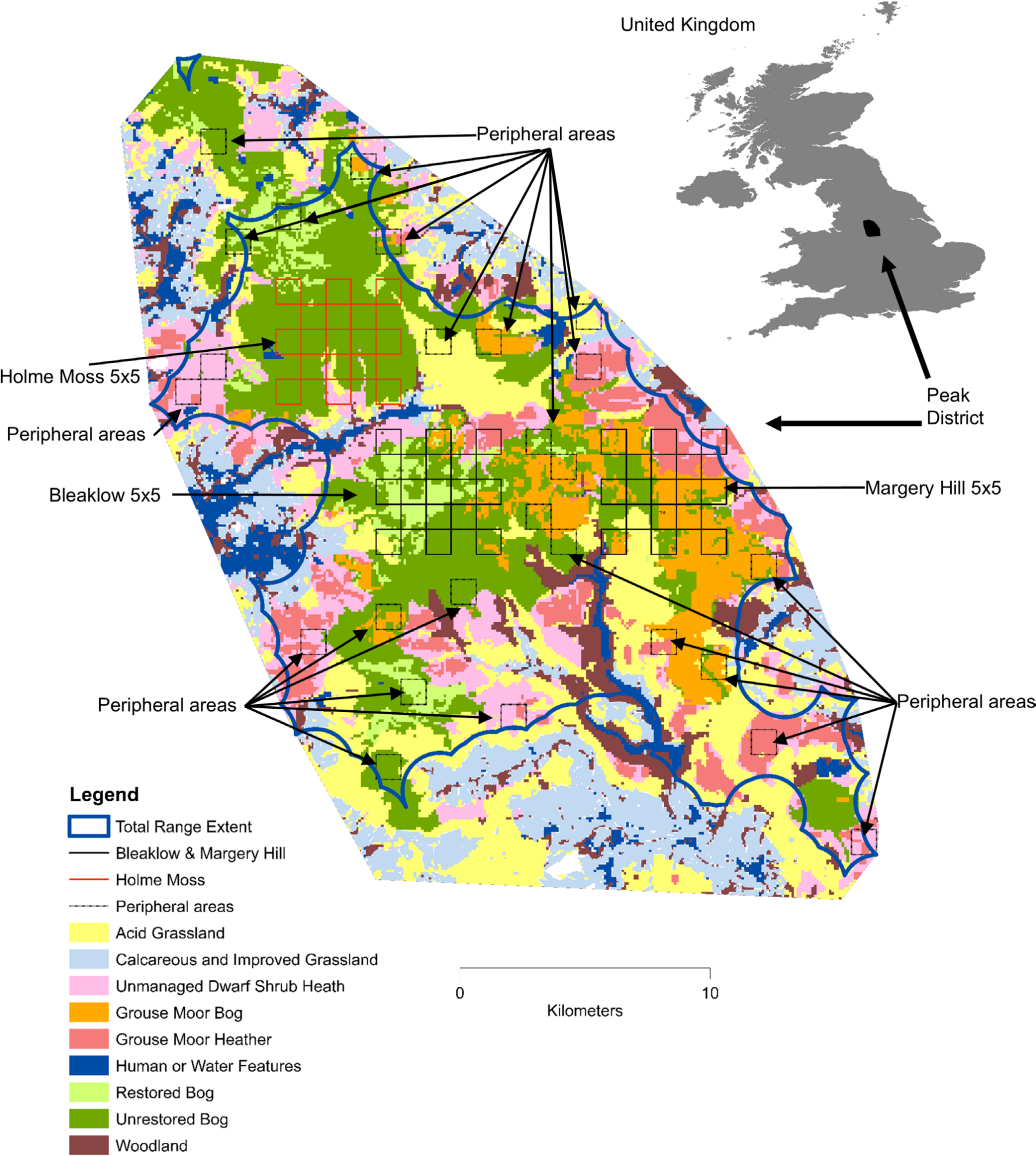
Map of study area reproduced from Bedson et al. (2022), figure 1. The locations of 10 years of BRC mountain hare records informed the minimum convex polygon, being the outer shape. The extent of hares for abundance projection was the alpha hull shape, shown by the blue line and also the survey areas. The survey transects are shown for Bleaklow and Margery Hill (5 × 5 black squares) surveyed consistently from 2017 to 2024. Holme Moss (5 × 5 red squares) was surveyed in 2018 and 2019. Peripheral areas (dotted squares) were surveyed in 2019. Legend shows habitat classes. Inset map shows location of Peak District in the United Kingdom. Peak District map origin is British National Grid Reference SK Easting 390,000 Northing 370,000.

Field work was conducted on upland habitats in the Peak District National Park, lying within the South Pennine Moors SAC (Figure 2). These uplands are underlain by acidic gritstone and shale rocks forming hills up to ~630 m elevation. The annual average temperature is 10.3°C, and precipitation is 1025 mm, creating a wet substrate on hill tops (UK Met Office 2020). The hills are covered with peat, up to 2 m deep (Anderson and Shimwell 1981). The study extent was informed by UK Biological Record Centre (BRC) mountain hare observations (see acknowledgments) for the period 1998–2018, eliciting 8666 records. From these, we mapped a minimum convex polygon of 610 km^2^ constituting the observed mountain hare range in our study area (Figure 2).

### 2.2 Habitat Classes

**FIGURE 3:**
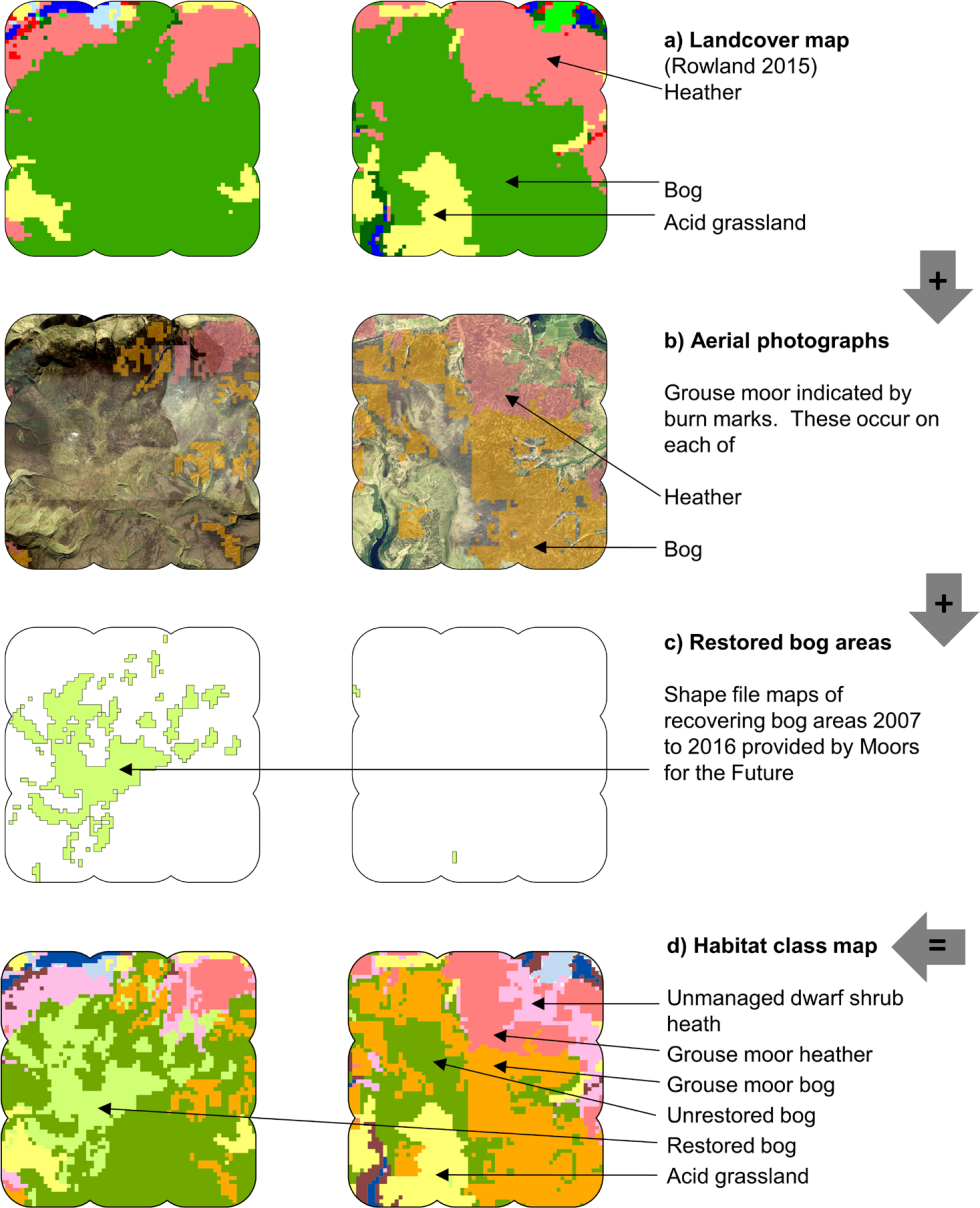
Step by step construction of habitat class map reproduced from Bedson et al. (2022), figure 2. Showing surveyed extent (5 × 5 km with 800 m buffer) with 1‐ha pixel, for each of Bleaklow (left) and Margery Hill (right) (British National Grid origin SK Easting 408,000 Northing 394,000). Map (a) shows landcover classification system of Rowland et al. (2017) which is used as starting point. Map (b) Aerial photographs are assessed and any with burn mark within any hectare denoted as either grouse moor bog or grouse heather, referencing the underlying landcover determined by Rowland et al. (2017). Map (c) Shape files provided by Moors for the Future, showing recovering bog areas which received treatment up to 2016. Map (d) The final map with all habitat classes pertinent to mountain hares. Any heather without burn mark is therefore regarded as unmanaged dwarf shrub heath.

We developed a habitat classification map by layering several data sources and mapping with a 1‐ha scale cell grid (i.e., 100 cells km^−2^)in ArcGIS (ArcMap 10.6.1, ESRI USA) (Figure 3). Habitat classes pertaining to mountain hare occupancy were acid grassland, upland dwarf shrub heath and wet upland blanket bog (Jackson 2000; Natural England 2005, 2019), with extent informed by the UK landcover map (Rowland et al. 2017). Other habitats had very few mountain hare records and were not included in analyses.

Acid grassland occurred at 300–550 m elevation, comprising a broad habitat type of calcifugous swards dominated by grasses ( *Festuca ovina* , *Nardus stricta* ), sedges, and herbs on lime‐deficient soils, pH < 5.5 (Jackson 2000; Rowland et al. 2017). In winter (when mountain hares were surveyed) grasses and bracken (*Pteridium*) were senescent; *Calluna* reached 80 cm in height, and *Juncus* and *Molinia* reached 120 cm in height (Stace 2010). These areas were used for sheep rearing.

Blanket bog comprised ombrotrophic wetlands supporting vegetation forming deep peat overlaying hill plateaus (Natural England 1993). *Eriophorum vaginatum* was dominant, with *Sphagnum* mosses and bog pools present (Anderson and Shimwell 1981; Natural England 2005; IUCN 2014; Rowland et al. 2017). These areas had been extensively eroded (Natural England 1993). We subdivided the blanket bog landcover area to “grouse moor bog”, “restored bog” and “unrestored bog” described below. Upland dry heath occupied lower slopes of moors on mineral soils or shallow peat areas, strongly dominated by *Calluna vulgaris‐Deschampsia flexuosa* and *C. vulgaris‐Vaccinium myrtillus* heath. (Anderson and Shimwell 1981; Elkington et al. 2001; Natural England 2005; Rowland et al. 2017) and we subdivided this to “grouse moor heather” or “unmanaged dwarf shrub heath” described below.

To identify grouse moor areas, we followed methods from Yallop et al. (2006) and assembled a mosaic of 1:500 scale aerial images dated for 2018 (Digimap 2019). Any 1‐ha cell showing a burn or mowed patch was designated as “grouse moor bog” or “grouse moor heather” depending on underlying landcover (Rowland et al. 2017). Grouse moors featured rotationally burned areas, shooting butts, grit trays, quad bike tracks and predator (corvid and mustelid) traps. “Grouse moor bog” at elevations 350–530 m were wet heath overlying deep peat with eroded gullies, *Calluna*, more *Eriophorum* spp. and mosses. “Grouse moor heather” at elevations 280–510 m, were drier areas with shallow peat, few gullies and extensive *Calluna* (Allen et al. 2016). Burned heather comprised different succession stages: suppressed (“pioneer”) heather, height to 15 cm; sub‐dominant heather, age to 10 + years, height ~15 cm, coverage ~40%; dominant heather, age up to 25 years, height ~30–120 cm coverage, 60 + % (Bardgett et al. 1995; Allen et al. 2016; Stace 2010; Whitehead et al. 2021). Also present were *Eriophorum*, *Sphagnum* and other mosses, cross‐leaved heather *Erica tetralix* , bell heather *Erica cinerea* , bilberry *Vaccinium myrtillus* and crowberry *Empetrum nigrum* (Bardgett et al. 1995; Whitehead et al. 2021). The Peak District was recorded with burns as 29% of total potential burn area and patch sizes 500–1000m^2^ (Allen et al. 2016).

The remaining heather area not grouse moor was classified as “Unmanaged dwarf shrub heath” at elevations 250–520 m including steep slopes and few gullies. This comprised mosaics of 70% dense/30% open heather, predominantly *Calluna* (Rowland et al. 2017), height to 120 cm (Bardgett et al. 1995; Stace 2010). There was no predator control and few sheep.

We identified “restored bog” from shapefiles provided by the conservation partnership “Moors for the Future” (Acknowledgements), designating their recovery work to 2016. These areas measured ~20km^2^, occurring at elevations 480–630 m and comprised previously degraded bare peat. From 2007 restoration efforts included gully blocking, fertiliser, liming, laying of jute textiles, reseeding, planting, spreading heather brash (Alderson et al. 2019). By 2016, this work achieved 75% vegetation cover (Alderson et al. 2019); much was in lush, verdant condition. Vegetation comprised a wide variety of moorland species which shifted frequently in composition over the space of a few metres, including *Calluna*, *Eriophorum* and *Sphagnum* spp., shrubs (e.g., *Erica tetralix* , *E. cinerea, Rubus chamemorus, Vaccinium mytrillus, Empetrum nigrum*), ferns (e.g., *Oreopteris limbosperma*, *Blechnum spicant* ), herbs (e.g., *Potentilla erecta* , *Viola palustris* , *Chamerion angustifolium* , *Galium saxatile* ), and mosses (e.g., *Hypnum jutlandicum* and *Polytrichum spp*). *Calluna* height was up to ~100 cm, winter grasses were senescent reaching heights ~30 cm (Stace 2010). The extensive networks of eroded gullies were revegetated and the water table was high (Alderson et al. 2019). There was no predator control practiced and sheep were fenced out.

The remaining bog areas were classed as “Unrestored bog” at elevations of 300–630 m. These had not historically deteriorated to the point of comprising bare peat; yet nonetheless appeared ecologically impoverished, that is, “unfavourable‐recovering” condition (Natural England 2019). They consisted mostly of extensive fields of *Eriophorum* spp. and *Molinia caerulea* grass, winter height ~30 cm, and some *Calluna* patches height ~100 cm (Stace 2010) with lower species diversity than restored bog areas. They featured eroded gullies, without gully blocking, as was the case for “restored bog”, they were drier with water run‐off. No predator control was practiced, and there were some sheep.

**FIGURE 4:**
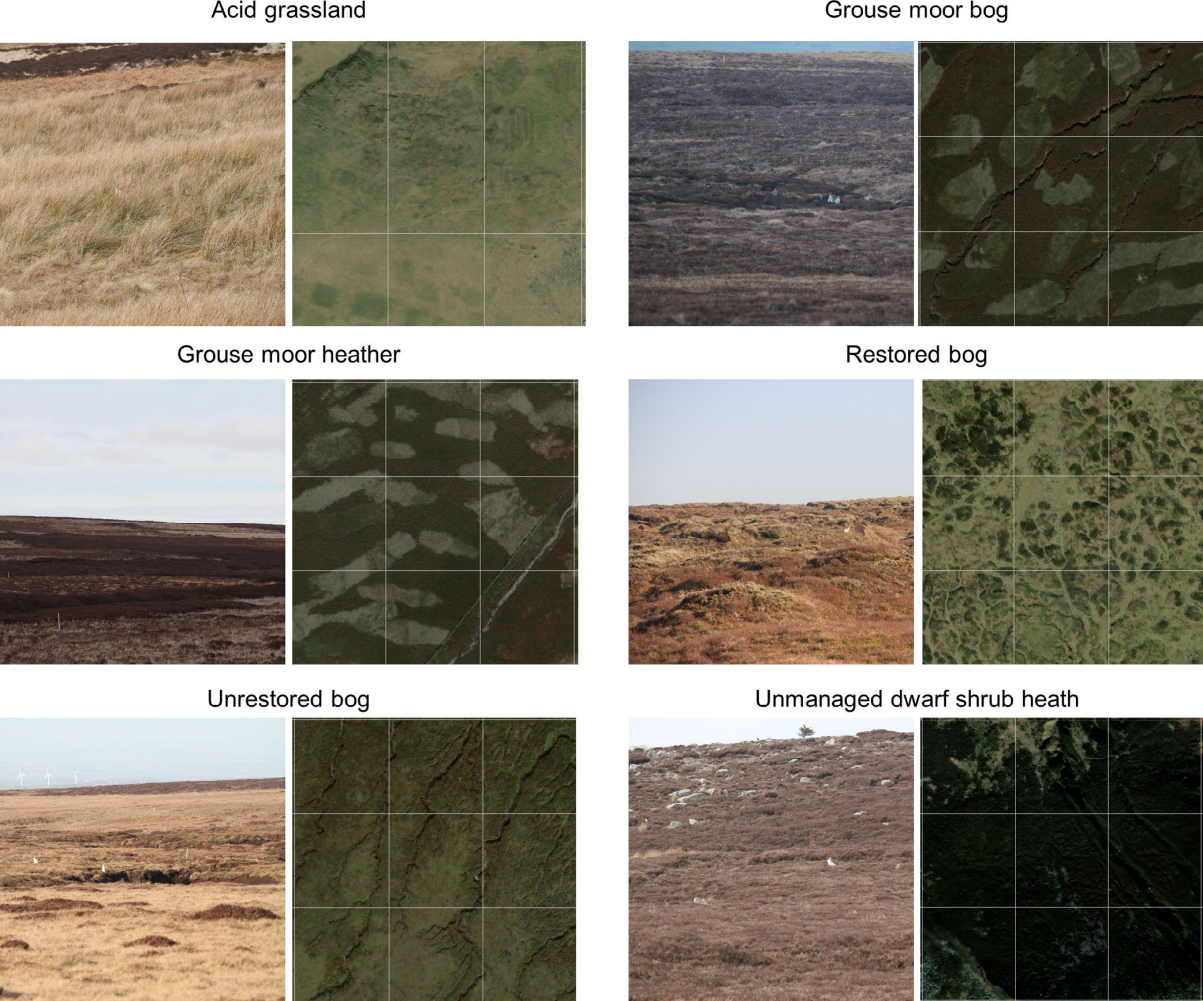
Photographs of each of the habitat classes reproduced from Bedson (2022), figure 3. For each habitat class, the left field photo is taken from the ground. The right side photos are aerial images at 300 m by 300 m with a 100 m fishnet grid overlain for scale; therefore, aerial photo scale as appears on page 1:4000 25 mm = 100 m. *Source*: ArcGIS ESRI “WorldImagery” downloaded 3 August 2021. Colours are natural, not enhanced. Note that each field photograph also contains an example mountain hare observation.

Ground and aerial photographs showing habitat classes appear in Figure 4. Table 1 lists vegetation communities. Habitat class data for hare observations, transect lengths and surveyed area size were then determined using “extract” function in the package “Raster” (Hijmans and van Etten 2012), superseded by “terra” (Hijmans 2024) within (R Core Team 2024).

**TABLE 1:** Ecosystems and habitat classes (reproduced from Bedson et al. (2022) Table 1) as used in this research and with the plant communities within these areas, described by the British National Vegetation Classification (NVC) (Jackson 2000; Elkington et al. 2001; Hall et al. 2004; Natural England 2021; Rowland et al. 2017; JNCC 2015).

Ecosystem	Habitat class	NVC category
Blanket bog	Restored bog	M1 and M2 *Sphagnum* bog‐pools M3 and M20 *Eriophorum* bog pools M4 *Carex rostrata* — *Sphagnum recurvum* mire M5 *Carex rostrata* — *Sphagnum squarrosum* mire M6 *Carex—Sphagnum* mires M9 *Carex rostrata* —*Calliergon cuspidatum/giganteum* mire M15 *Scirpus cespitosus—Erica tetralix* wet heath M16 *Erica tetralix—Sphagnum compactum* wet heath M19 *Calluna—Eriophorum* blanket mires
	Unrestored bog	As for Restored bog
	Grouse moor bog	As for Restored bog
Upland dry heath	Grouse moor heather	H1 *Calluna—Festuca* heath H8 *Calluna—Ulex* heath H9 *Calluna—Deschampsia* heath H10 *Calluna– Erica* heath H12 *Calluna—Vaccinium* heath H18 *Vaccinium—Deschampsia* heath M19 *Calluna—Eriophorum* blanket mires
	Unmanaged dwarf shrub heath	H1 *Calluna—Festuca* heath H8 *Calluna—Ulex* heath H9 *Calluna—Deschampsia* heath H10 *Calluna– Erica* heath H12 *Calluna—Vaccinium* heath H18 *Vaccinium—Deschampsia* heaths
Acid grassland	Acid grassland	U1 *Festuca ovina—Agrostis capillari—Rumex acetosella* grassland U2 *Deschampsia flexuosa* grassland U4 *Festuca ovina—Agrostis capillaris—Galium saxatile* grassland U5 *Nardus stricta—Galium saxatile* grassland U6 *Juncus squarrosus—Festuca ovina* grassland *W16 Quercus* spp. *– Betula* spp. *– Deschampsia flexuosa* woodland (for bracken)

### 2.3 Surveys

Transect surveys were conducted from 2017 to 2024 upon 26 Ordnance Survey maps alternating 1 km grid squares within 5 × 5km square arrays, central to Bleaklow and Margery Hill; both observable areas effectively measuring 40.4km^2^ within the wider 610km^2^ of the Peak District extent (Figure 2). To enable subsequent comparison of density by habitat class (acid grassland; grouse moor bog; grouse moor heather; restored bog; unrestored bog; unmanaged dwarf shrub heath) their spatial distributions were recorded in Bedson et al. (2022), and in this now subsequent paper, regarded as unchanged, evidenced by field observations and satellite image download (Landsat 8 scene Path203/Row023 10th May 2024 USGS: www.earthexplorer.usgs.gov) compared with the grouse moor shape previously described.

Separately, a survey was made of a wider area, including a 5 × 5km site on Holme Moss in 2018 and 2019 and also in 2019 an additional 26 × 1 km randomly selected squares (peripheral areas). Lacking additional staff, these areas were not surveyed in any other years; although the 2019 surveys enabled a Peak District‐wide population estimate for that year, subsequently referred to.

Survey transects followed each 1km^2^ square perimeter, guided by GPS (Garmin 64MapST,15 m accuracy) and were conducted during daylight hours from January through April. The survey schedule randomised squares the first year, maintaining the same schedule each year as logistics allowed. Each side of the square was surveyed once, looking on both sides of the transect, walking very slowly and taking 2–5 h. Surveyors scanned ahead with binoculars every 200 m to locate hares or groups of hares in the undulating terrain. Only observations made while walking along the transect line were included in the analysis.

Mountain hare observations were made using standard distance sampling methods, recording date time, grid reference, cluster size distance to hare (Nikon ProStaff7i laser range finder, accuracy 1 m), and angle (compass and angle board) (Buckland et al. 2001). Potential double counts for observations were discounted.

The preceding study (Bedson et al. 2022) recorded hare behaviour to investigate if hare activity (hiding, flushing etc) influenced the detection process. We continued recording behaviour; however, as the preceding study concluded this was not influential, it was not analysed again.

Surveys were conducted under similar conditions for comparable previous studies in clear weather with wind speeds < 20mph (e.g., Newey et al. 2018). We assumed stronger winds did not influence hare detections (e.g., Flux 1962), but caused difficulties holding the laser range finder steady. No surveys were conducted with snow present.

### 2.4 Distance Modelling

For Bleaklow and Margery Hill, mountain hare observations were attributed to the habitat class on which the animal was first seen (as represented in Figure 1). Previous analysis (Bedson et al. 2022) showed field measurement errors might misallocate up to 2.7% of observations to an incorrect class, which we continued to accept as a tolerable systematic error.

We analysed our data with DISTANCE v.7.3 (Thomas et al. 2010), using different data filtering and model selections, the same as Bedson et al. (2022). We assessed different truncation distances and bin widths. We compared detection models with three key functions: uniform, half‐normal, and hazard rate, with cosine or polynomial expansion terms (Buckland et al. 2001 47; Williams and Thomas 2007). We assessed the suitability of assumptions and models using histograms, quantile‐quantile plots, *χ* ^2^ goodness of fit statistics, and the fit of the detection function close to the transect line g(0). We compared and sought simple models with few parameters, lower AIC values between models using the same data selection, higher *χ* ^2^ goodness of fit statistics and lower detection probability *cv* values (Buckland et al. 2001).

From 2017 to 2021, 2010 observations were recorded, and from 2022 to 2024 there were 613 new mountain hare observations; these were combined together as one data set. Perpendicular distance data from 2017 to 2021 were previously analysed with a data filter, by truncating observations seen further than 520 m from the transect lines, and the hazard rate model with 3 parameters, using a global detection function when stratifying (Bedson et al. 2022). We reassessed these choices of data filter, model suitability and stratification. With the newly combined data set, having ~600 additional records with new distance to object measurements, model evaluation and parameter values for the detection function changed slightly. The highest *χ* ^2^goodness of fit statistic was 0.99, with a truncation at 500 m, the hazard rate model and estimating detection probability *p* = 0.19, P cv = 0.04 (Table S1). However, repeating the model used in the previous study (Bedson et al. 2022) that is, truncation at 520 m and hazard rate model, this continued to report a very high *χ* ^2^goodness of fit statistic (0.95), *p* = 0.18, P cv = 0.04, that is, very similar. Therefore, for consistency, minimising any analytical change to previous reporting, this data filter (520 m truncation) and model selection (hazard rate) were retained. We investigated whether analysis would be better served with a stratified detection function: this provided AIC = 28965.64, slightly lower compared to global detection function AIC = 29010.54. However, some stratified detection probabilities had wide confidence intervals (e.g., acid grassland cv = 0.28) and there was insufficient sample size for unmanaged dwarf shrub heath (*n* = 43), so we retained the global detection function, consistent with Bedson et al. (2022).

**FIGURE 5:**
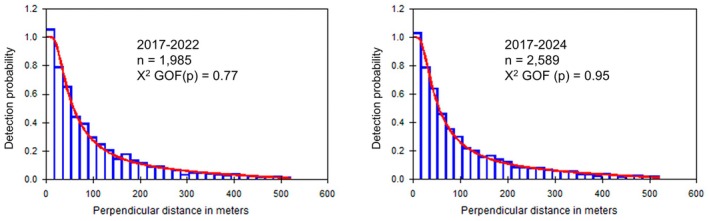
Comparison of probability density functions for the two data sets obtained from Software Distance, showing sample sizes and Chi‐*p* goodness‐of‐fit test values.

Therefore, with the addition of the new distance to object measurements from 2022 to 2024, the detection probability function did slightly change its shape, and this was the only and unavoidable change to modelling compared to Bedson et al. (2022). Table 2 reports these differences, showing an increase (improvement) in the Chi‐square goodness of fit test, a slight decrease in detection probability and effective strip width, and a slight decrease (improvement) in the coefficient of variation. Figure 5 compares the two probability density function histograms for 2017–2021 and 2017–2024.

**TABLE 2:** Comparison of probability density function parameter estimates 2017–2021 versus 2017–2024 based on data truncated at 520 m, and the hazard rate model fitted with 3 adjustment terms.

	Survey Period 2017–2021 (*n* = 1985)			Survey Period 2017–2024 (*n* = 2589)		
	Estimate	% CV	df	Estimate	% CV	df
m	3			3		
LnL	−11,071			−14,502		
AIC	22,148			29,011		
Chi‐*p*	0.77			0.95		
f(0)	0.010467	0.040	1982	0.010754	0.038	2586
*p*	0.183	0.040	1982	0.179	0.038	2586
ESW (m)	95.5	0.040	1982	92.9	0.038	2586

Abbreviations: % CV = coefficient of variation; AIC = Akaike Information Criterion; Chi‐*p* Probability of chi‐squared goodness‐of‐fit test; df = degrees of freedom; ESW(m) = effective strip width in metres; f(0) = 1/u = value of probability density function at zero; LnL = Likelihood value; m = number of parameters in the model; *n* = sample size; *p* = probability of observing an object in a defined area.

The change in probability density function provided a slight difference to previous parameter estimates for 2017–2021 reported in Bedson et al. (2022), shown for completeness in (Table S2).

For stratification, following Bedson et al. (2022), the global detection function was applied, with encounter rate, cluster size and density varying by strata in three ways: (1) by year, pooled within each year, without habitat information; (2) by habitat class, mean values for all eight years together; (3) by habitat and by years, that is, 6 habitats over 8 years = 48 strata. Each stratification analysis used the same data, allocating transects, transect distances and observations to the different strata definitions. Parameter estimates were compared, with 95% confidence intervals. Density comparisons between pairwise combinations of the strata of habitat classes for 2017–2024 were made using the t‐statistic, which applies the Satterthwaite approximation, accounting for unequal sample sizes (Buckland et al. 2001: 84–86) and considers the lack of independence of data arising from using a common detection function between strata. We evaluated significance with a Bonferroni corrected *p*‐value and also calculated effect sizes (Field et al. 2012: 57–58).

Potential overall population trends were analysed with generalised linear models (GLMs). The distance sampling pooled annual “density” estimates were considered as the response variable, and “year” as the only predictor variable. The distance sampling density estimates stratified by habitat class were also modelled with year as the only predictor, enabling comparisons with the pooled estimate. To facilitate model choices, density values were rounded to integers, assessing Gaussian and Poisson (link = identity) GLMs and comparing model quality with AIC which reported: Gaussian = 49.50; Poisson (link = identity) = 46.04 (Smith et al. 2020). Thus, the Poisson GLM (link = identity) provided the lowest AIC in all cases, except acid grassland, and so for consistency was adopted for all. We considered the density value of any year might unduly influence the model if residuals assessed with Cook's distance computed > 1 (Field et al. 2012: 269). We considered model gradient estimates as population trends. All years and habitat classes showed decreasing gradients, so we divided the 2024 density estimate by the gradient to forecast time to extinction, assuming continuing rates of decline. Statistical analysis was conducted with the “stats” package and graphs produced with “ggplot2” (Wickham 2016) in base R software version 4.3.3 (R Core Team 2024).

Abundance was calculated for the study sites. Abundance was extrapolated to the wider Peak District based on density proportions from Bedson et al. (2022) when in 2019 Bleaklow and Margery Hill reported 22.9 hares km^−2^ and wider peripheral areas 6.1 hares km^−2^ (i.e., 27% of the former).

We held the broad assumption that this proportion remained the same, applying 27% from the Bleaklow and Margery Hill density estimate for 2024 to calculate abundance for the wider Peak District.

Maps were produced for all geo‐referenced hare observation locations for each year using ArcGIS (ESRI USA).

## 3 Results

Surveys from 2017 to 2024 covered 961 km and with observation data truncated at 520 m perpendicular distance, we recorded 2589 detections (Table 3). Over the survey period, the encounter rate decreased from 2.5 hares km^−1^ during 2017 to 1.2 hares km^−1^ in 2024. Cluster size reduced from 1.18 individuals per encounter in 2017 to 1.04 in 2024, that is, fewer, smaller groups of hares. Estimated density decreased from 15.9 hares km^−2^ (95% CI 10.4–24.4 hares km^−2^) in 2017 by 58% to 6.7 hares km^−2^(3.9–11.5 hares km^−2^) in 2024 (Table 3).

**TABLE 3:** 2017–2024 parameter estimates with probability detection function from 2017 to 2024 data set.

Survey Period 2017–2024													
Years	*n*	*L*	*n*/*L*	*n*/*L* CV	*n*//*L* LCL	*n//L* UCL	*K*	E (s)	E (s) CV	D̂̂	D̂̂ CV	D̂̂ LCL	D̂̂ UCL
2017	304	120.9	2.5	0.20	1.7	3.8	26	1.18	0.02	15.9	0.21	10.4	24.4
2018	504	121.6	4.1	0.10	3.3	5.2	26	1.15	0.01	25.6	0.11	20.1	32.2
2019	401	112.5	3.6	0.14	2.6	4.8	26	1.13	0.02	21.7	0.15	15.7	29.7
2020	402	123.1	3.3	0.25	1.9	5.5	26	1.06	0.01	18.6	0.26	10.9	31.6
2021	374	120.8	3.1	0.18	2.1	4.4	26	1.13	0.01	18.8	0.18	12.8	27.3
2022	246	121.3	2.0	0.22	1.2	3.1	26	1.06	0.01	11.6	0.22	7.3	18.4
2023	214	121.3	1.8	0.23	1.0	2.8	26	1.09	0.01	10.3	0.23	6.4	16.7
2024	144	119.9	1.2	0.26	0.7	2.0	26	1.04	0.01	6.7	0.26	3.9	11.5

*Note:* D̂̂ is calculated with probability density function f(0) = 0.010754 and f(0) cv = 0.0379.

Abbreviations: cv = parameter coefficient of variation; D̂̂ = density estimate km^−2^; E(s) = mean cluster size; K = number of transects; L = line length km; LCL and UCL = 95% confidence intervals; *n* = encounters.

**FIGURE 6:**
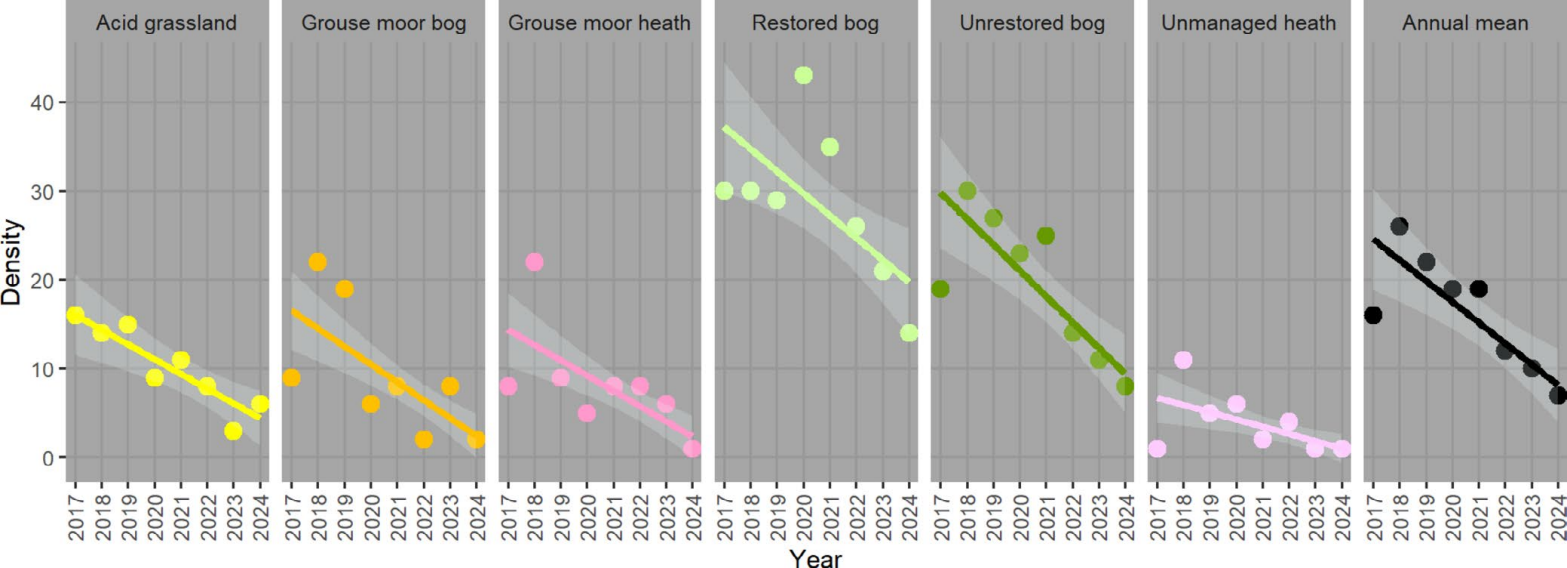
Density trend estimates of mountain hares km^−2^ for Bleaklow and Margery Hill combined, for 2017–2024 by habitat class. X‐axis is year; y‐axis is density (Habitat strata density estimate as reported Table S3; Annual mean density estimate as reported Table 3). Each plot facet shows a different habitat class, with density point estimates as dots with colours as Figure 7. Trajectories are plotted with Poisson GLM (link = identity), showing 95% confidence intervals in lighter grey that is, representing gradients of Table 5. In every year, there were more mountain hares on restored bog than on any other habitat class.

For the 8 years, parameter estimates by habitat class are shown in Table 4 and detailed annual parameter estimates by habitat class are shown in Table S3. Density estimates and trajectories by habitat class are represented in Table 5 and Figure 6, showing restored bog with the highest mean density (27.9 hares km^−2^; 95% CI 22.9–35.2), followed by unrestored bog (19.3 hares km^−2^; 95% CI 16.7–23.1). Land managed for grouse shooting had fewer hares: grouse moor bog (9.3 hares km^−2^; 95% CI 7.3–12.0); grouse moor heather (8.3 hares km^−2^; 95% CI 5.4–12.5). Acid grassland was similar (9.0 hares km^−2^; 95% CI 6.0–14.8). Unmanaged dwarf shrub heath had the lowest mean hare density (4.0 hares km^−2^; 95% CI 2.3–6.7).

**TABLE 4:** 2017–2024 parameter estimates by habitat class with probability detection function from the 2017–2024 data set.

Habitats	*n*	*L*	*n*/*L*	*n*/*L* CV	*n*//*L* LCL	*n*//*L* UCL	*K*	E (s)	E (s) CV	D̂̂	D̂̂ CV	D̂̂ LCL	D̂̂ UCL
Acid grassland	92	71.5	1.3	0.22	0.9	2.1	57	1.30	0.04	9.0	0.23	6.0	14.8
Grouse moor bog	343	215.7	1.6	0.12	1.2	2.0	136	1.09	0.01	9.3	0.12	7.3	12.0
Grouse moor heath	101	76.1	1.3	0.20	0.9	2.0	38	1.16	0.03	8.3	0.20	5.4	12.5
Restored bog	757	160.7	4.8	0.10	3.9	5.8	90	1.10	0.01	27.9	0.11	22.9	35.2
Unrestored bog	1253	383.4	3.3	0.07	2.9	3.8	189	1.10	0.01	19.3	0.08	16.7	23.1
Unmanaged heath	43	64.2	0.7	0.26	0.4	1.1	73	1.11	0.04	4.0	0.27	2.3	6.7

*Note:* D̂̂ is calculated with probability density function f(0) = 0.010754 and f(0) cv =0.0379.

Abbreviations: cv = parameter coefficient of variation; D̂̂ = density estimate km^−2^; E(s) = mean cluster size; *K* = number of transects; *L* = line length km; LCL & UCL = 95% confidence intervals; *n* = encounters.

**TABLE 5:** Poisson (link = identity) GLM model estimates of mountain hare density km^−2^ by year, for Bleaklow and Margery Hill combined for 2017–2024 and by habitat class informed by Table S3.

	Coefficients								
	Estimate	Standard Error	*t* value	*p* value	Significant	Cook's distance > 1 (Outlier)	2017–2024 change	2024 Density	# Projected years to extinct
Annual mean (Intercept)	4753.5	1204.72	3.95	0.0001	*				
Annual mean (Year)	−2.3	0.60	−3.93	0.0001	*	2017	−58%	6.7	3
Acid grassland (Intercept)	3364.3	938.52	3.59	0.0003	*				
Acid grassland (Year)	−1.7	0.46	−3.58	0.0004	*		−62%	6.2	4
Grouse moor bog (Intercept)	4073.7	848.86	4.80	0.0000	*	2017			
Grouse moor bog (Year)	−2.0	0.42	−4.79	0.0000	*		−73%	2.4	1
Grouse moor heath (Intercept)	3489.0	804.94	4.33	0.0000	*				
Grouse moor heath (Year)	−1.7	0.40	−4.33	0.0000	*	2018, 2024	−86%	1.2	1
Restored bog (Intercept)	5064.4	1638.39	3.09	0.0020	*				
Restored bog (Year)	−2.5	0.81	−3.07	0.0021	*		−55%	13.5	5
Unrestored bog (Intercept)	5895.6	1313.85	4.49	0.0000	*				
Unrestored bog (Year)	−2.9	0.65	−4.47	0.0000	*	2017	−56%	8.3	3
Unmanaged heath (Intercept)	1651.6	543.33	3.04	0.0024	*				
Unmanaged heath (Year)	−0.8	0.27	−3.04	0.0024	*	2017	−33%	0.8	1

*Note:* Coefficients = Intercept and Year (gradient) estimates with standard error, *t*‐value and *p*‐value with significance at the < 0.05 level. Cook's distance reports if any year's density estimate is an influential outlier to the model. 2017–2024 shows density estimate % change from 2017 to 2024. 2024 is point estimate. #project years to extinct divides the 2024 density estimate by the model gradient coefficient estimate, calculating how many years the population would endure at the 2017–2024 trend.

The Poisson GLM of annual density values showed a significant decrease in mountain hare density km‐^2^ from 2017 to 2024, both pooled and for each of the habitat strata (Table 5, Figure 6). Of habitat classes, grouse moor heather showed the steepest decrease (86%). Unmanaged dwarf shrub heath showed the least decrease (33%). Cook's distance assessments showed density estimates from 2017 were unduly influential model outliers for the overall annual density GLM and for those of grouse moor bog, unrestored bog and unmanaged dwarf shrub heath. For grouse moor heath two outliers acted in opposite directions (2018 particularly high; 2024 low). With the hypothetical assumption that model trajectories would continue, the population was arithmetically calculated to become extinct in approximately three to five years, enduring for the longest on restored bog.

Pairwise t‐tests based on habitat class density estimates (Table 4) showed restored blanket bog having significantly higher hare density than all other habitat classes. Unrestored bog density was significantly higher than grouse moor bog and grouse moor heather. Grouse moor bog, grouse moor heather, and acid grassland were similar to each other. Unmanaged dwarf shrub heath was significantly lowest of all habitat classes (Table 6).

**TABLE 6:** Pairwise *t*‐tests of habitat class estimates for 2017–2024.

Strata 1	Strata 2	D̂̂ difference	SE Diff	*t* stat	df *t* stat	*p*	Significant	Bonferonni‐corrected significant	Effect size
Acid grassland	Grouse moor bog	−0.3	0.002	0.14	110.36	0.888			0.01
Acid grassland	Grouse moor heath	0.7	0.002	0.27	106.60	0.786			0.03
Acid grassland	Restored bog	−18.8	0.003	5.39	182.51	0.000	*	**	0.40
Acid grassland	Unrestored bog	−10.3	0.002	4.16	150.72	0.000	*	**	0.34
Acid grassland	Unmanaged dwarf shrub heath	4.9	0.002	2.17	98.96	0.032	*		0.22
Grouse moor bog	Grouse moor heath	1.0	0.002	0.51	85.48	0.607			0.06
Grouse moor bog	Restored bog	−18.5	0.003	6.05	154.85	0.000	*	**	0.48
Grouse moor bog	Unrestored bog	−10.0	0.001	5.52	483.48	0.000	*	**	0.25
Grouse moor bog	Unmanaged dwarf shrub heath	5.0	0.001	3.40	226.58	0.000	*	**	0.23
Grouse moor heath	Restored bog	−19.5	0.003	5.91	159.39	0.000	*	**	0.47
Grouse moor heath	Unrestored bog	−11.0	0.002	5.02	127.24	0.000	*	**	0.44
Grouse moor heath	Unmanaged dwarf shrub heath	4.2	0.001	2.14	74.00	0.035	*		0.25
Restored bog	Unrestored bog	−8.5	0.003	2.71	185.21	0.007	*	**	0.20
Restored bog	Unmanaged dwarf shrub heath	−23.0	0.003	7.82	146.32	0.000	*	**	0.64
Unrestored bog	Unmanaged dwarf shrub heath	−15.3	0.001	8.55	364.07	0.000	*	**	0.45

*Note:* D̂̂ difference (hares km^−2^) subtracts Strata 2 D̂̂ from Strata 1 D̂̂. A positive value indicates Stratum 1 is larger; a negative value means Stratum 2 is larger. SE is the standard error of D̂̂ difference. Values are assessed with the Satterthwaite t test, reporting t statistic and degrees of freedom. Asterisk indicates *p*‐value significance and also when applying Bonferroni within‐cohort correction at the 0.0033 level. Effect size is calculated with Cohen's d and considered as *r* = 0.10 (small); *r* = 0.30(medium); *r* = 0.50(large).

**FIGURE 7:**
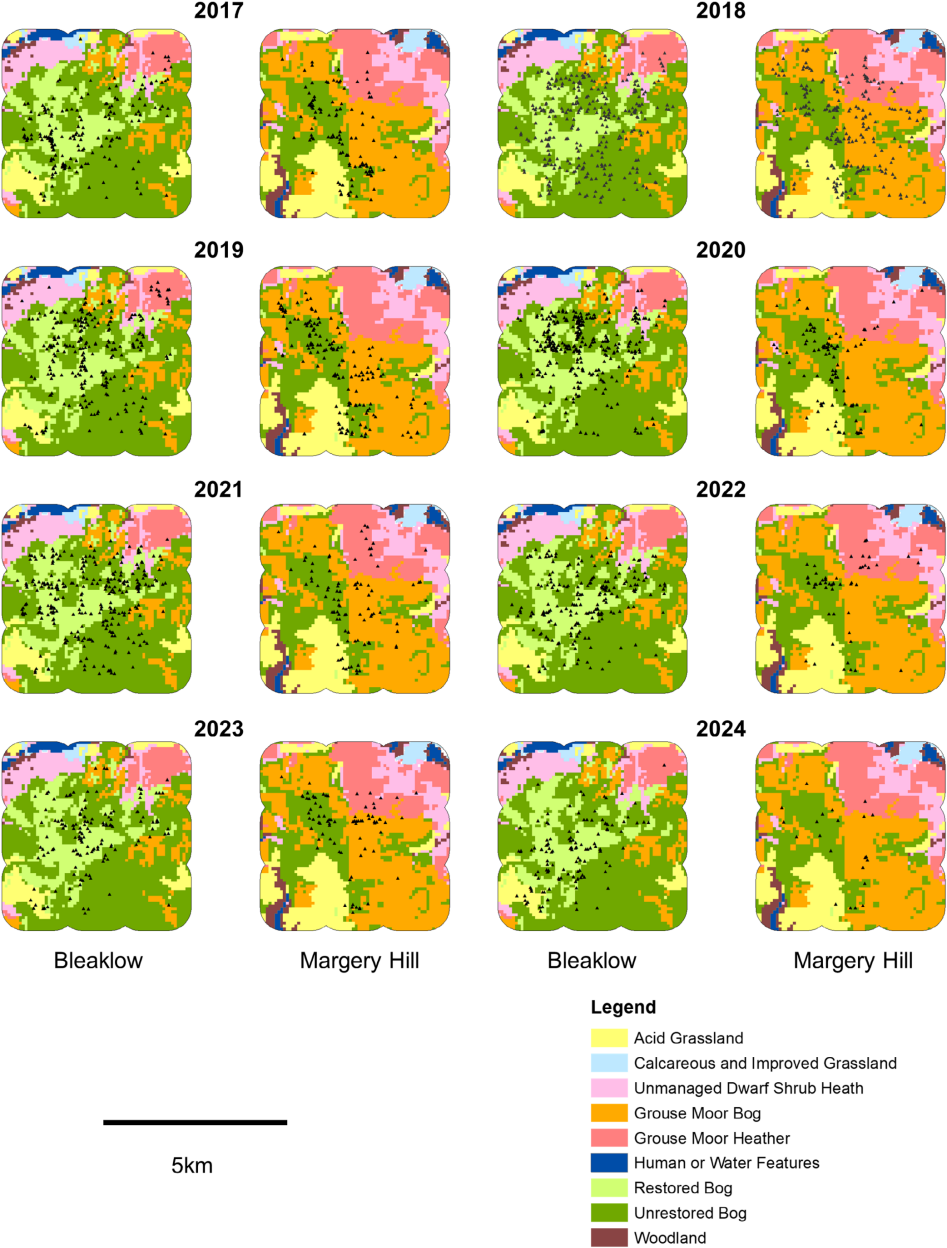
Maps showing locations of mountain hare detections (black triangles) on Bleaklow and Margery Hill 2017–2024 with habitat types shown, colours as per legend. Visible surveyed range was 40.4km^2^ for each of Bleaklow and Margery Hill.

Figure 7 shows maps of hare observations by habitat by year. From 2017 to 2024, surveys on Bleaklow had a higher number of encounters widespread across the entire 5 × 5km survey area, consistently on restored and unrestored bog; some presence on grouse moor heath until 2020; inconsistent presence on acid grassland. Margery Hill surveys saw fewer encounters, substantial on grouse moor heath and grouse moor bog in 2018, then diminishing; consistent encounters on unrestored bog to 2023. Yet in 2024, Margery Hill observations were reduced to just 22; those were mostly on the central area of grouse moor bog and unrestored bog habitat.

For Bleaklow and Margery Hill (80.8km^2^), as density decreased (Table 3), abundance was calculated to have decreased from 1287 (95% CI 840–1971) during 2017 to 542 (95% CI 315–929) hares during 2024. Considering the wider Peak District held 27% of mountain hare density as compared to Bleaklow and Margery Hill (Bedson et al. 2022), the density estimate for 2024 for the wider Peak District reported abundance declining from 3562 individuals (95% CI 2291–5624) (Bedson et al. 2022) to 1038 (95% CI 604–1765) individuals (Table 7).

**TABLE 7:** Abundance estimate for Bleaklow and Margery Hill for 2024, extrapolated to the Peak District.

	Bleaklow and Margery Hill	Remaining area	Total peak district
Density	6.7	1.8	
Density LCL	3.9	1.0	
Density UCL	11.5	3.0	
Area km^2^	80.8	276.7	357.5
Abundance	541	497	1038
Abundance LCL	315	289	604
Abundance UCL	929	836	1765

## 4 Discussion

Mountain hare density was monitored from 2017 to 2024 using the same protocols and methods in the two highest density areas of the Peak District, examining population trends and on different habitat classes. Distance sampling analysis with this eight‐year data set reported a detection probability with a very low coefficient of variation (P cv = 0.04) and an annual density estimate D cv = 0.11–0.26, making this one of the most reliable survey regimens for this species for the purpose of reporting narrow confidence intervals, enabling detection of statistically significant differences in absolute density over time. For example, Newey et al. (2018) reported mark/recapture P cv = 0.14–3.72 (therefore stratified and not global value) and D cv = 0.14–0.37, though with one year of survey effort. With detected group sizes being close to one, that is, single individuals, encounter rate variation represents the main parameter that influences the overall density estimate.

The main finding of this monitoring was a statistically significant seven‐year decline of mountain hare density on Bleaklow and Margery Hill by 58% from 15.9 (95% CI: 10.4–24.4) to 6.7 (95% CI: 3.9–11.5) individuals km^−2^. There were statistically significant declines in all habitat classes, differing in gradient. Acid grassland showed a consistent gradual decline. Trajectories declined the most for grouse moor heath and second most for grouse moor bog. Restored bog densities fluctuated, yet with an overall decline. Unrestored bog showed a generally consistent decline. Unmanaged dwarf shrub heath declined the least. The combined population for Bleaklow and Margery Hill for 2017 reduced from 1287 individuals in 2017 to 542 individuals in 2024. In the same period, the total population for the Peak District was calculated as reducing from 3562 to 1038 individuals. Assuming constant rates of decline, the mountain hare population on Bleaklow and Margery Hill would calculate as becoming extinct within three to five years.

Retrospectively, we reviewed the validity of this survey regimen for detecting either an impending extinction or a longer‐term trend of cyclicity. Buckland and Johnson (2017) outline ideal requirements for wildlife monitoring: representative sampling locations; sufficient sample sizes (effort); sufficient detections must be achieved; survey timing should ensure less population variation and be repeated at the same time each year. We consider these criteria have been fully met, although with the exception of geographic extent. Survey locations are representative of the wide geography of the Peak District: evidence from 2019 showed Bleaklow and Margery Hill hosting relatively high mountain hare densities (noting Watson et al. (1973) suggesting low = 0–5; moderate = 5–20; high = > 20). However, we do not know the density trends for other colonies of mountain hares, for example, Holme Moss, Kinder Scout and these areas warrant investigation to provide stronger population trajectory evidence. Otherwise, this monitoring regimen is altogether consistent with the ideal requirements of Buckland and Johnson (2017). Our effort and sample sizes are in excess of the requirements for conventional distance sampling (needing ~70 observations). The survey occasion is repeated in winter, assuming only adult hares, that is, a stable population. Analytical protocols were consistent with previous reporting (Bedson et al. 2022).

When analysing long‐term or volatile time series data of abundance estimates, Buckland et al. (2004) recommend using generalised additive models (GAM) which moderate or smooth the density index. However, when the data trend is shorter or stable, linear regression or GLMs are regarded as a reasonable approaches, with the advantage they provide summarised recognisable estimate values (e.g., gradient, *p*‐value). When assessing monitoring results, Buckland and Johnson (2017) suggest that schemes may require “settling down” because of lower sample sizes obtained in the first year, which might lead to wider parameter estimate fluctuations or confidence intervals; hence, to set as baseline a subsequent year with a larger data set, which may increase precision. Indeed, our GLM analysis showed some 2017 values as outliers to the subsequent trend, which was a consistent decline from 2018 to 2024. Referring instead to 2018 as the baseline year would show a yet steeper decline of density estimate, i.e. (2018 = 25.6) to (2024 = 6.7) a decline of 74%. Yet overall, the study period of eight years is still relatively short and Buckland et al. (2004) question the wisdom of extrapolating predictions from a short timespan empirical model into the future.

Hence, we contemplate whether the decline of Peak District mountain hares to extinction is plausible. Mountain hares have occupied this location since the 1870s, yet now at very low densities, and one speculates such declines happened before (e.g., severe winter 1962–1963, Mallon et al. 2003). Mountain hares have persisted at similar low densities in the west of Scotland for many hundreds of years (Watson and Hewson 1973; Hamill et al. 2006). However, small populations may succumb to weather or disease remarkably quickly, such as a group of ~400 mountain hares on the island Lilla Karlsö, Sweden, altogether disappearing in the severe winter of 1976/77 (Angerbjorn and Hjernquist 1984). With the extrapolated abundance for the Peak District at ~1000 mountain hares, the small absolute population size and ongoing declining trajectory, these factors contribute to extinction risk, known to be higher for animals with small body sizes or small ranges (Newsome et al. 2019).

We then consider possible reasons for the recent Peak District mountain hare decline. The most likely vector may be parasite‐mediated population cyclicity, with crashes of up to 90% and periodicities varying from 4 to 15 years in Scotland (Newey et al. 2007). We cannot say whether the eight‐year decline we identified represents part of such a cycle, with a rebound to be expected, or whether the decline will be ongoing.

Risks to small populations may be precipitated by factors which are hard to identify and confounding: land use change, habitat fragmentation, roadkill, over harvesting, climate change, genetics, disease and population cyclicity (Mills 2013), which we consider in turn. Bleaklow and Margery Hill show a spectrum of land uses which have stayed the same in nature and extent from 2017 to 2024. Intensive grouse moor management contributes to a Calluna dominated landscape with reported lower mountain hare densities. Evidence for harvesting of mountain hares appears anecdotal (Bedson 2022: 241, 250, 268); neither is it not known whether pre‐emptive removal of hares to mitigate against louping ill virus occurs in the Peak District, as suggested for Scotland (Watson and Wilson 2018). By contrast, the restored blanket bog, originally conceived for carbon sequestration, appears to provide superior floral diversity i.e. a broader range of food and shelter resource, beneficial for mountain hares. During surveys in restored bog locations, we also observed increasing numbers of human tourists post‐Covid, with mountain hares appearing slightly human habituated i.e. less evasive movement than upon grouse moors. These differences of density by habitat resemble mechanisms articulated by Mills (2013) where habitat loss may contribute to local decreases in species abundance, with transient increases to neighbouring fragments i.e. a displacement or “crowding on the ark” effect. Roadkill of mountain hares was reportedly high 2017–2022 (Bedson 2022), and may have reduced, concurrent with overall population decline. Climate change is expected to increase mountain hare vulnerability to predation and reduce the distribution of mountain hare populations (Zimova et al. 2020; Bedson et al. 2021); with six of the ten warmest years since 1884 occurring between 2014 and 2023 (Kendon et al. 2024) concurrent to the mountain hare decline trajectory. A review of genetic indices of hares across the northern Hemisphere, showed the mountain hares of Scotland having low diversity, which may have propagated to their progeny in the Peak District (Bedson 2022: 209). Disease has not been evidenced.

Therefore, we are unclear about what has caused this decline and what may be done to arrest it. Veterinary autopsies of mountain hare carcasses, measuring levels of *Trichostronglyus retortaeformis* intestinal parasites, might inform population cyclicity. If mountain hares were listed for England on Schedule 5 of the 1981 Wildlife and Countryside Act, this may provide better protection against harvest. Further blanket bog restoration may be conducive. Translocation of mountain hares from Scotland might not be appropriate without knowing whether the Peak District mountain hares represent a sink population. To better understand these matters, telemetry data from GPS‐collared hares could be used to inform a known fate model, and responses of mountain hares to space, habitat, land use, and humans (e.g., Marchand et al. 2014).

This small population of mountain hares in the Peak District, regarded as a Victorian era conservation success, has uncertain longevity, threatened by local human pressures and climate change. Their prospective disappearance implies adverse consequences for the uplands ecosystem: loss of sizable herbivores, naturally fertilising and spreading seeds, whose carcasses provide substantial biomass for carnivores and raptors, for carrion insects and the small mammals and birds that prey upon them, and also nutrients (phosphorous, nitrogen, calcium) for soils and vegetation. In effect, mountain hares are a keystone species. Their continued persistence, an extension of the *Lepus timidus scoticus* population, may represent potentially important genetic distinctiveness. They represent upland habitat quality, are a visible manifestation of wild mammal population dynamics responding to human pressures and climate change, and reflect conservationists' capacity to support biodiversity. Similar present‐day translocations attract substantial investment to facilitate species recovery. Peak District mountain hares are a reminder to ensure species are reintroduced into enduringly benign natural environments.

We are continuing this monitoring regimen of mountain hares in the Peak District of the upland hills covered in this research. In future years, we anticipate providing more results from these distance sampling surveys, ascertaining whether the decline of the mountain hare in England continues, stabilises, or rebounds.

## Author Contributions

**Carlos P. E. Bedson:** conceptualization (lead), data curation (lead), formal analysis (lead), investigation (lead), methodology (lead), software (lead), validation (equal), visualization (lead), writing – original draft (lead), writing – review and editing (equal). **Katherine Walsh:** formal analysis (supporting), investigation (supporting), validation (equal), writing – review and editing (supporting). **Humphrey Q. P. Crick:** conceptualization (supporting), formal analysis (supporting), validation (equal), writing – review and editing (supporting).

## Disclosure

Note temporary reviewer Url: https://datadryad.org/stash/share/V1oGt8eo1yPMc8aoHPnoJIj‐UYYZKvs49NISB_SOIOQ.

## Conflicts of Interest

The authors declare no conflicts of interest.

## Supporting information


Data S1.

## Data Availability

Distance sampling observation and measurement data are available from the Dryad Digital Repository https://doi.org/10.5061/dryad.wwpzgmstj.
