# Supplementary material for: Seven Year Decline of Mountain Hare Abundance in the Peak District, England
Source: Ecol Evol. 2025 Mar 17;15(3):e71131. doi: 10.1002/ece3.71131 (PMC11917130; doi:10.1002/ece3.71131)
Supplement: Supplementary file 1 — Data S1. [file ECE3-15-e71131-s001.docx]

**Supplementary Information**

Table S1

Range of candidate models based on all data for Bleaklow and Margery Hill, pooled, 2017 to 2024. n = number of observations; Model (key) = Key function with series expansion; AIC = Akaike Information Criterion; ΔAIC = delta AIC value within comparable data selections; χ2 GOF (p)= chi-square goodness of fit *P*-value; P = detection probability function; P *cv* = detection probability coefficient of variation. We chose to use data truncated at 520m with the hazard-rate model and polynomial, for all analyses.

| **Data selection** | **N** | **Model (key)** | **# para** | **AIC** | **ΔAIC** | **χ2 GOF (p)** | **P** | **P *cv*** |
| --- | --- | --- | --- | --- | --- | --- | --- | --- |
| Truncate at 520m | 2589 | Uniform + cosine | 1 | 30183.32 | 1172.78 | 0.00 | 0.51 | 0.00 |
|  |  | Uniform + poly | 3 | 29715.62 | 705.08 | 0.00 | 0.40 | 0.01 |
|  |  | Half-normal + cosine | 3 | 29175.70 | 165.16 | 0.00 | 0.25 | 0.02 |
|  |  | Half-normal + Hermite | 1 | 29688.43 | 677.89 | 0.00 | 0.36 | 0.01 |
|  |  | Hazard rate + cosine | 3 | 29014.02 | 3.48 | 0.91 | 0.18 | 0.03 |
|  |  | Hazard rate + poly | 3 | 29010.54 | 0.00 | 0.95 | 0.18 | 0.04 |
| Truncate at 500m | 2581 | Uniform + cosine | 3 | 29130.14 | 276.22 | 0.00 | 0.31 | 0.01 |
|  |  | Uniform + poly | 0 | 0.00 | 0.00 | 0.00 | 0.00 | 0.00 |
|  |  | Half-normal + cosine | 3 | 29008.18 | 154.26 | 0.00 | 0.26 | 0.02 |
|  |  | Half-normal + Hermite | 1 | 29509.12 | 655.20 | 0.00 | 0.37 | 0.01 |
|  |  | Hazard rate + cosine | 3 | 28855.95 | 2.03 | 0.98 | 0.19 | 0.03 |
|  |  | Hazard rate + poly | 3 | 28853.92 | 0.00 | 0.99 | 0.19 | 0.04 |
| Truncate at 480m | 2569 | Uniform + cosine | 3 | 28874.82 | 246.68 | 0.00 | 0.31 | 0.01 |
|  |  | Uniform + poly | 3 | 29241.77 | 613.63 | 0.00 | 0.41 | 0.01 |
|  |  | Half-normal + cosine | 3 | 28767.17 | 139.03 | 0.00 | 0.26 | 0.02 |
|  |  | Half-normal + Hermite | 1 | 29247.46 | 619.32 | 0.00 | 0.38 | 0.01 |
|  |  | Hazard rate + cosine | 3 | 28630.26 | 2.12 | 0.65 | 0.20 | 0.04 |
|  |  | Hazard rate + poly | 3 | 28628.14 | 0.00 | 0.75 | 0.19 | 0.04 |

Table S2 Comparison of 2017-2021 parameter estimates when obtained with probability detection function using either 2017-2021 or 2017-2024 data sets. n = encounters; L = line length km; K = number of transects; E(s) = mean cluster size; ­D = density estimate km^−2^; cv = parameter coefficient of variation; LCL & UCL = 95% confidence intervals. ­D is calculated with probability density function f(0) and f(0) cv. (Buckland et al., 2001, 84,85). Density for the 2017-2021 data set calculated with f(0)=0.010754 and f(0) cv =0.0407 ; for 2017-2024 with f(0)=0.010467 and f(0) cv =0.0379 . Encounter rate parameters are identical. Cluster size parameters marginally different because of distance size biased regression calculation. Density estimates for 2017 to 2021 are therefore ~3% lower when using the 2017-2021 dataset, as compared to using the 2017-2024 dataset.

| Survey Period 2017-21 | | | |  |  |  |  |  |  |  |  |  |  |  |
| --- | --- | --- | --- | --- | --- | --- | --- | --- | --- | --- | --- | --- | --- | --- |
| **Years** |  |  | |  |  |  |  |  |  |  |  |  |  |  |
|  | n | L | n/L | | n/L CV | n//L LCL | n//L UCL | K | E (s) | E (s) CV | D̂̂ | D̂̂ CV | D̂̂ LCL | D̂̂ UCL |
| 2017 | 304 | 120.9 | 2.5 | | 0.20 | 1.7 | 3.8 | 26 | 1.18 | 0.02 | 15.5 | 0.21 | 10.1 | 23.9 |
| 2018 | 504 | 121.6 | 4.1 | | 0.10 | 3.3 | 5.2 | 26 | 1.14 | 0.01 | 24.7 | 0.11 | 19.6 | 31.5 |
| 2019 | 401 | 112.5 | 3.6 | | 0.14 | 2.6 | 4.8 | 26 | 1.13 | 0.01 | 21.1 | 0.15 | 15.4 | 29.0 |
| 2020 | 402 | 123.1 | 3.3 | | 0.25 | 1.9 | 5.5 | 26 | 1.05 | 0.01 | 17.9 | 0.26 | 10.6 | 30.8 |
| 2021 | 374 | 120.8 | 3.1 | | 0.18 | 2.1 | 4.4 | 26 | 1.13 | 0.01 | 18.3 | 0.18 | 12.6 | 26.8 |
|  |  |  |  | |  |  |  |  |  |  |  |  |  |  |
|  |  |  |  | |  |  |  |  |  |  |  |  |  |  |
| Survey Period 2017-24 | | |  | |  |  |  |  |  |  |  |  |  |  |
| **Years** |  |  |  | |  |  |  |  |  |  |  |  |  |  |
|  | n | L | n/L | | n/L CV | n//L LCL | n//L UCL | K | E (s) | E (s) CV | D̂̂ | D̂̂ CV | D̂̂ LCL | D̂̂ UCL |
| 2017 | 304 | 120.9 | 2.5 | | 0.20 | 1.7 | 3.8 | 26 | 1.18 | 0.02 | 15.9 | 0.21 | 10.4 | 24.4 |
| 2018 | 504 | 121.6 | 4.1 | | 0.10 | 3.3 | 5.2 | 26 | 1.15 | 0.01 | 25.6 | 0.11 | 20.1 | 32.2 |
| 2019 | 401 | 112.5 | 3.6 | | 0.14 | 2.6 | 4.8 | 26 | 1.13 | 0.02 | 21.7 | 0.15 | 15.7 | 29.7 |
| 2020 | 402 | 123.1 | 3.3 | | 0.25 | 1.9 | 5.5 | 26 | 1.06 | 0.01 | 18.6 | 0.26 | 10.9 | 31.6 |
| 2021 | 374 | 120.8 | 3.1 | | 0.18 | 2.1 | 4.4 | 26 | 1.13 | 0.01 | 18.8 | 0.18 | 12.8 | 27.3 |

Table S3 Stratified distance sampling parameter estimates by habitat class by year 2017-2024. n = encounters; L = line length km; K = number of transects; E(s) = mean cluster size; ­D = density estimate km^−2^; cv = parameter coefficient of variation; LCL & UCL = 95% confidence intervals. ­D is calculated with probability density function f(0) and f(0) cv. (Buckland et al., 2001, 84,85). Density for calculated with f(0)=0.010467 and f(0) cv =0.0379. Habitat / year AG = Acid grassland; GMB = Grouse moor bog; GMH = Grouse moor heath; RB= Restored bog; UB = Unrestored bog; UH = Unmanaged dwarf shrub heath.

| Habitat / year | n | L | n/L | n/L CV | n//L LCL | n//L UCL | K | E (s) | E (s) CV | D̂̂ | D̂̂ CV | D̂̂ LCL | D̂̂ UCL |
| --- | --- | --- | --- | --- | --- | --- | --- | --- | --- | --- | --- | --- | --- |
|  |  |  |  |  |  |  |  |  |  |  |  |  |  |
| AG17 | 11 | 8.3 | 1.3 | 0.61 | 0.3 | 5.3 | 7 | 2.31 | 0.25 | 16.5 | 0.66 | 3.6 | 59.6 |
| GMB17 | 41 | 27.0 | 1.5 | 0.31 | 0.8 | 2.9 | 17 | 1.10 | 0.06 | 9.0 | 0.31 | 4.1 | 15.4 |
| GMH17 | 12 | 9.8 | 1.2 | 0.31 | 0.5 | 2.9 | 5 | 1.28 | 0.12 | 8.4 | 0.34 | 3.2 | 17.3 |
| RB17 | 93 | 19.8 | 4.7 | 0.32 | 2.3 | 9.4 | 11 | 1.18 | 0.03 | 29.8 | 0.32 | 13.2 | 53.9 |
| UB17 | 145 | 47.1 | 3.1 | 0.17 | 2.2 | 4.4 | 24 | 1.15 | 0.03 | 19.0 | 0.17 | 11.9 | 24.5 |
| UH17 | 2 | 8.7 | 0.2 | 1.12 | 0.0 | 1.8 | 9 | 1.00 | 0.00 | 1.2 | 1.12 | 0.1 | 8.8 |
| AG18 | 20 | 8.7 | 2.3 | 0.55 | 0.7 | 8.1 | 7 | 1.16 | 0.10 | 14.3 | 0.56 | 3.6 | 45.3 |
| GMB18 | 102 | 27.2 | 3.8 | 0.12 | 2.9 | 4.8 | 18 | 1.10 | 0.03 | 22.2 | 0.13 | 15.0 | 25.7 |
| GMH18 | 32 | 9.6 | 3.3 | 0.23 | 1.6 | 7.0 | 4 | 1.25 | 0.06 | 22.4 | 0.24 | 9.8 | 40.5 |
| RB18 | 99 | 19.9 | 5.0 | 0.20 | 3.2 | 7.7 | 12 | 1.11 | 0.03 | 29.7 | 0.20 | 17.0 | 41.4 |
| UB18 | 239 | 47.1 | 5.1 | 0.10 | 4.1 | 6.3 | 23 | 1.11 | 0.02 | 30.3 | 0.11 | 22.4 | 25.5 |
| UH18 | 12 | 8.9 | 1.3 | 0.62 | 0.4 | 5.0 | 10 | 1.57 | 0.11 | 11.4 | 0.63 | 2.7 | 37.6 |
| AG19 | 19 | 8.0 | 2.4 | 0.50 | 0.7 | 7.6 | 7 | 1.18 | 0.08 | 15.1 | 0.51 | 4.1 | 43.0 |
| GMB19 | 80 | 25.1 | 3.2 | 0.23 | 1.9 | 5.2 | 17 | 1.10 | 0.03 | 18.9 | 0.24 | 10.2 | 27.8 |
| GMH19 | 12 | 9.1 | 1.3 | 1.04 | 0.1 | 14.2 | 5 | 1.23 | 0.01 | 8.7 | 1.04 | 0.7 | 82.8 |
| RB19 | 86 | 18.3 | 4.7 | 0.19 | 3.0 | 7.3 | 10 | 1.16 | 0.03 | 29.3 | 0.20 | 16.7 | 41.0 |
| UB19 | 196 | 43.6 | 4.5 | 0.14 | 3.3 | 6.1 | 23 | 1.12 | 0.02 | 27.1 | 0.15 | 17.6 | 33.3 |
| UH19 | 8 | 8.1 | 1.0 | 0.73 | 0.2 | 4.4 | 9 | 1.01 | 0.11 | 5.4 | 0.74 | 1.0 | 22.2 |
| AG20 | 14 | 8.9 | 1.6 | 0.39 | 0.6 | 4.0 | 7 | 1.09 | 0.05 | 9.2 | 0.40 | 3.2 | 21.0 |
| GMB20 | 25 | 27.6 | 0.9 | 0.25 | 0.5 | 1.5 | 17 | 1.15 | 0.05 | 5.6 | 0.26 | 2.9 | 8.6 |
| GMH20 | 10 | 10.0 | 1.0 | 0.42 | 0.3 | 3.7 | 4 | 1.00 | 0.00 | 5.4 | 0.42 | 1.3 | 17.4 |
| RB20 | 150 | 20.0 | 7.5 | 0.30 | 3.8 | 14.7 | 11 | 1.07 | 0.02 | 43.2 | 0.31 | 19.6 | 75.9 |
| UB20 | 193 | 47.5 | 4.1 | 0.24 | 2.4 | 6.7 | 23 | 1.04 | 0.01 | 22.7 | 0.25 | 12.2 | 33.9 |
| UH20 | 10 | 8.9 | 1.1 | 0.48 | 0.4 | 3.2 | 9 | 1.00 | 0.00 | 6.0 | 0.48 | 1.8 | 15.6 |
| AG21 | 11 | 8.4 | 1.3 | 0.56 | 0.4 | 4.5 | 8 | 1.50 | 0.12 | 10.6 | 0.57 | 2.7 | 32.8 |
| GMB21 | 37 | 26.9 | 1.4 | 0.20 | 0.9 | 2.1 | 16 | 1.09 | 0.05 | 8.1 | 0.22 | 4.5 | 11.3 |
| GMH21 | 13 | 9.8 | 1.3 | 0.46 | 0.4 | 4.6 | 5 | 1.16 | 0.14 | 8.3 | 0.49 | 2.2 | 24.7 |
| RB21 | 116 | 19.7 | 5.9 | 0.24 | 3.4 | 10.2 | 10 | 1.11 | 0.05 | 35.1 | 0.25 | 18.2 | 54.8 |
| UB21 | 192 | 47.1 | 4.1 | 0.14 | 3.0 | 5.5 | 23 | 1.15 | 0.03 | 25.2 | 0.15 | 16.4 | 31.2 |
| UH21 | 5 | 10.5 | 0.5 | 0.91 | 0.1 | 2.8 | 7 | 0.81 | 0.17 | 2.1 | 0.95 | 0.3 | 13.9 |
| AG22 | 3 | 8.7 | 0.3 | 0.39 | 0.1 | 0.8 | 7 | 4.60 | 0.53 | 8.5 | 0.66 | 0.8 | 83.4 |
| GMB22 | 12 | 27.2 | 0.4 | 0.24 | 0.2 | 0.9 | 17 | 0.99 | 0.06 | 2.3 | 0.35 | 1.1 | 4.8 |
| GMH22 | 12 | 9.1 | 1.3 | 0.32 | 0.5 | 3.1 | 5 | 1.15 | 0.09 | 8.2 | 0.34 | 3.4 | 19.4 |
| RB22 | 93 | 20.4 | 4.6 | 0.18 | 3.0 | 6.8 | 12 | 1.08 | 0.02 | 26.5 | 0.19 | 17.5 | 40.3 |
| UB22 | 122 | 48.6 | 2.5 | 0.22 | 1.5 | 3.9 | 20 | 1.04 | 0.02 | 14.0 | 0.22 | 8.8 | 22.3 |
| UH22 | 4 | 7.0 | 0.6 | 0.79 | 0.1 | 2.8 | 9 | 1.15 | 0.20 | 3.5 | 0.86 | 0.6 | 18.7 |
| AG23 | 5 | 8.4 | 0.6 | 0.80 | 0.1 | 3.3 | 7 | 1.00 | 0.23 | 3.2 | 0.83 | 0.5 | 17.8 |
| GMB23 | 34 | 27.7 | 1.2 | 0.35 | 0.5 | 2.5 | 17 | 1.17 | 0.05 | 7.7 | 0.35 | 3.7 | 15.0 |
| GMH23 | 8 | 9.1 | 0.9 | 0.27 | 0.4 | 1.8 | 5 | 1.21 | 0.24 | 5.7 | 0.37 | 2.2 | 12.7 |
| RB23 | 72 | 19.9 | 3.6 | 0.29 | 1.9 | 6.7 | 12 | 1.07 | 0.02 | 20.8 | 0.29 | 10.9 | 39.5 |
| UB23 | 94 | 49.1 | 1.9 | 0.28 | 1.0 | 3.4 | 24 | 1.07 | 0.02 | 11.0 | 0.28 | 6.1 | 19.8 |
| UH23 | 1 | 6.8 | 0.1 | 1.14 | 0.0 | 1.2 | 9 | 1.00 | 0.00 | 0.8 | 1.14 | 0.9 | 6.4 |
| AG24 | 9 | 8.4 | 1.0 | 0.40 | 0.2 | 7.0 | 9 | 1.07 | 0.18 | 6.2 | 0.40 | 1.7 | 40.3 |
| GMB24 | 12 | 26.6 | 0.5 | 0.30 | 0.2 | 0.8 | 17 | 1.00 | 0.00 | 2.4 | 0.30 | 1.2 | 4.5 |
| GMH24 | 2 | 9.1 | 0.2 | 0.60 | 0.0 | 1.0 | 5 | 1.00 | 0.00 | 1.2 | 0.60 | 0.2 | 5.5 |
| RB24 | 48 | 20.3 | 2.4 | 0.30 | 1.2 | 4.5 | 12 | 1.06 | 0.02 | 13.5 | 0.33 | 6.9 | 26.2 |
| UB24 | 72 | 48.3 | 1.5 | 0.23 | 0.9 | 2.4 | 24 | 1.04 | 0.02 | 8.3 | 0.23 | 5.1 | 13.7 |
| UH24 | 1 | 6.8 | 0.1 | 1.07 | 0.0 | 1.1 | 9 | 1.00 | 0.00 | 0.8 | 1.07 | 0.1 | 5.9 |
